# Killing Clothes Lice by Holding Infested Clothes Away from Hosts for 10 Days to Control Louseborne Relapsing Fever, Bahir Dah, Ethiopia

**DOI:** 10.3201/eid2502.181226

**Published:** 2019-02

**Authors:** Stephen C. Barker, Dayana Barker

**Affiliations:** University of Queensland, Brisbane, Queensland, Australia (S.C. Barker);; University of Queensland School of Veterinary Sciences, Gatton, Queensland, Australia (D. Barker)

**Keywords:** Phthiraptera, Pediculus humanus, louseborne relapsing fever, lice, clothes lice, infested clothes, barrels, Borrelia recurrentis, bacteria, vector-borne infections, zoonoses, London School of Hygiene and Tropical Medicine, World War I, World War II, Ethiopia

## Abstract

Louseborne relapsing fever (LBRF) was once a cosmopolitan disease, but it now occurs only in the Horn of Africa. Recent cases in refugees to Europe made LBRF topical again. Crowded boarding houses and church dwellings in Ethiopia are analogous to the crowded air-raid shelters of World War II. Thus, we might learn from experiments the London School of Tropical Hygiene and Medicine conducted during World War II. When the vector of *Borrelia recurrentis* (*Pediculus humanus* lice) was held away from the host for 10 days, 100% of nymphal and adult lice starved to death and 100% of eggs did not hatch. We hypothesize that holding infested clothes away from hosts in plastic shopping bags will kill enough lice to control LBRF in Ethiopia. Owning 2 sets of clothes might be useful; 1 set might be held in a plastic shopping bag for 10 days to kill lice and their eggs.

Louseborne relapsing fever (LBRF), which is caused by the spirochete *Borrelia recurrentis*, once had a cosmopolitan distribution but is now endemic only to countries in the Horn of Africa (Ethiopia, Sudan, South Sudan, and Somalia) (*1*,*2*). A recent spate of cases of LBRF in refugees and migrants to Europe from the Horn of Africa underlines the point that LBRF is endemic to and prevalent in this part of Africa (*3*–*19*). LBRF is one of the top 10 reasons why persons visit healthcare clinics in Ethiopia (*20*). Thus, LBRF is a substantial burden on the healthcare system and, moreover, a cause of substantial illness and death, especially in day laborers, street children, and yekolotemaries (live-in church students) in cities such as Bahir Dah in the Amhara Region of the highlands of Ethiopia (altitude 1,800 m).

There have been substantial outbreaks of LBRF in Bahir Dah (*21*–*25*). Moreover, some patients with undiagnosed febrile disease come to healthcare clinics in Bahir Dah and elsewhere in Ethiopia because of LBRF and other louse-associated diseases, such as louseborne epidemic typhus (LBET) (caused by *Rickettsia prowazekii*) and trench fever (caused by *Bartonella quintana*).

LBRF has been eradicated from all regions of the world, except the Horn of Africa, by activities that kill clothes lice (*Pediculus humanus*), which have also been referred to as body lice. Some of these activities have been organized public health interventions in communities, including mass delousing by steaming infested clothes with Stammers Serbian barrels, which were developed during World War I (*26*), and are still used today in Ethiopia (Figure 1) and elsewhere. Other activities include parents delousing clothes of their children by washing clothes in hot water (>60°C).

**Figure 1:**
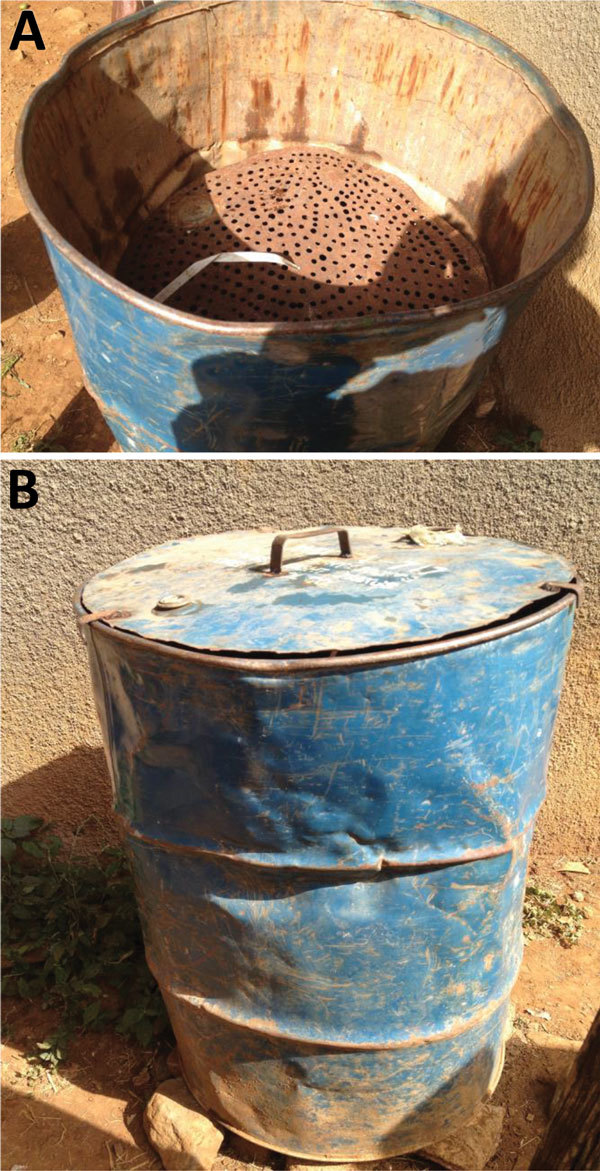
Wood-fired steaming barrel from a healthcare center in the highlands of Ethiopia that is used to kill clothes lice and their eggs during outbreaks of louseborne relapsing fever. A) Top view; B) front view showing lid. This 200-L barrel is modeled on the Stammers Serbian barrels.

Some of these activities have been part of intentional schemes to reduce prevalence and intensity of louseborne pathogens, but most of these activities have been unintentional from the point of view of LBRF—programs to improve the cleanliness and social status of persons and their families. Lice are abhorred by most persons, and infestation with clothes lice is invariably associated, right or wrong, with low socioeconomic status. However, regardless of the motivation, activities that reduce prevalence and intensity of infestation with clothes lice have been successful in reducing prevalence and intensity of louseborne pathogens, particularly since World War II (*27*,*28*).

In the context of louse-associated diseases, other authors have emphasized the benefits of washing bodies and clothes (*22*,*29*); it has been reported that clothes lice prefer a dirty shirt to a clean shirt (*30*). Washing clothes in hot water (>60°C) for >10 min kills clothes lice and their eggs (*30*). However, immersion of clothes in water heated to 60°C by a charcoal or wood fire is costly and time-consuming for most persons in Ethiopia.

We have studied head lice and community-level and individual-level strategies to control head lice in Brisbane, Queensland, Australia, and elsewhere for many years (>20 years for S.C.B. and 5 years for D.B.) (*31*–*33*). When we washed the hair of infested children with soap or shampoo or with water alone, we observe deaths of only a few adult lice; nymphal instars 1, 2, and 3; and eggs of lice. It appears that head lice close their spiracles when immersed in water; lice have to be submerged in water for >19 hours to be killed (*34*). Our experience with head lice led us to wash cloth that was infested with clothes lice and eggs of clothes lice only with soap or water. This cloth was naturally infested cloth in several countries and cloth infested in the laboratory with lice and eggs from the Barker isolate of the Culpepper strain of clothes lice.

Our experience with clothes lice was similar to our experience with head lice; washing infested clothes in warm or cold water for 30 min with or without soap or washing detergent kills few lice and their eggs, although it results in clean clothes, clean lice, and clean eggs. Attacking lice and their eggs in clothes and blankets of persons is a simple way to reduce the prevalence and intensity of louseborne pathogens, such as *B. recurrentis* (cause of LBRF) and *R. prowazekii* (cause of LBET).

Attacking lice in clothes and blankets was the first public health intervention that stopped a budding epidemic of an arthropod-associated disease: the systematic treatment of clothes of many of the 1 million inhabitants of Naples, Italy, with pyrethrum powder and DDT powder in 1943. The account of this delousing campaign in air-raid shelters and other places in Naples has been reported (*35*–*37*). This delousing intervention stopped a budding epidemic of LBET. However, systematic treatment of clothes of all of or even a large group of the inhabitants of Bahir Dah with pyrethrum powder, DDT powder, or a synthetic pyrethroid is not ideal because of its immediate and ongoing cost.

After studying data from the London School of Hygiene and Tropical Medicine (London, UK) during World War II, we conceived a strategy that might reduce the number and severity of outbreaks of LBRF in day laborers, street children, and yekolotemaries in Bahir Dah. If successful, this strategy might be adapted to other situations in the Horn of Africa and elsewhere, where louse-associated diseases still occur. The crowded boarding houses (Figure 2) that house day laborers during the heavy rainy season and crowded dwellings of yekolotemaries of Bahir Dah and other cities and towns in Ethiopia are in many ways analogous to the crowded air-raid shelters and trenches that gave respite from bombs in World War II; the boarding houses and church dwellings in Bahir Dah give respite from torrential rains during the rainy season (June–September). Thus, we might learn from the scientific and epidemiologic investigations of medical entomologists during World War II, such as Patrick Buxton, James Busvine and Major H. S. Leeson of the London School of Tropical Hygiene and Medicine, who found that when clothes lice were held away from their host for 7 days at 10°C, or for 10 days at any temperature, 100% of nymphal and adult lice starved and died. Moreover, when clothes lice were held at <19°C, 100% of eggs did not hatch (died).

**Figure 2:**
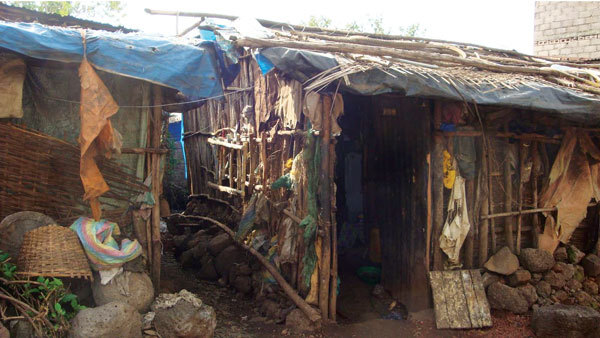
Typical boarding house in Bahir Dah, Ethiopia, in which many day laborers sleep at night during the heavy rainy season (June–September; Table) because it is too wet to sleep outdoors. At other times of the year when it does not rain so heavily and so often, day laborers in Bahir Dah tend to sleep outdoors and, thus, do not have to pay for lodging in boarding houses. Image courtesy of S.C. Barker.

**Table:** Temperature and precipitation data for Bahir Dah, Amhara Region, Ethiopia*

Variable	Month												Year
	Jan	Feb	Mar	Apr	May	Jun	July	Aug	Sep	Oct	Nov	Dec	
Daily temp., °C (range)	18.5 (8–29)	20 (9–31)	21.5 (11–32)	22.5 (13–32)	22.5 (13–32)	15 (13–29)	19 (13–26)	19 (13–25)	19 (12–26)	19.5 (12–27)	19 (10–28)	18 (8–28)	20.5 (11.3–28.8)
No. rainy days	1	1	2	3	10	18	28	28	20	10	3	1	125
Monthly rainfall, mm	2	2	12	28	80	205	396	375	211	87	12	6	1,416
*All values are averages. Data were obtained from the World Meteorological Organization (https://www.wmo.int/pages/index_en.html). The altitude of Bahir Dah is 1,800 m. During the heavy rain season (June–September), it rains on most days: June, 18/30 d; July 28/31 d; August, 28/31 d; and September, 20/30 d. Temp., temperature.													

Our hypothesis, drawn from these data, is that holding infested clothes away from hosts in plastic shopping bags will kill enough clothes lice to control LBRF in Bahir Dah and elsewhere in Ethiopia. Owning 2 sets of clothes would enable 1 set to be held in a plastic shopping bag for 10 days to starve to death adult and nymphal lice; even 7 days away from the host will kill most of the lice and eggs. This hypothesis might be tested in Bahir Dah and elsewhere in Ethiopia and the Horn of Africa.

It is encouraging that in at least 1 part of Ethiopia, the catchment of the Gambo General Rural Hospital in southwestern Ethiopia, the number of cases of LBRF seems to have decreased during 1997–2007 (*38*). The reason(s) for this decrease are unclear, although louse infestation seems to have become less intense in the patients attending the Gambo General Rural Hospital (*38*). We can expect a decrease in cases of LBRF in Bahir Dah and other towns and cities in Ethiopia if the prevalence and intensity of clothes lice can be substantially reduced because this has been the experience worldwide. It is not known how low the prevalence and intensity of infestation with clothes lice need to be to prevent periodic outbreaks of LBRF in day laborers, street children, and yekolotemaries in Bahir Dah (*22*–*25*). However, low-tech and simple public health strategies aimed at killing the lice and eggs that infest these 3 populations offer the prospect of reducing LBRF in these groups (*22*–*25*).

In this study, we use the name clothes lice instead of body lice for *P. humanus* lice because the name clothes lice or clothing lice (*39*) has at least 2 advantages over the name body lice. First, clothes lice is more accurate because, in our experience, most lice on infested persons will be found in the clothes at any time. Second, these lice go to the skin to feed. Once they have fed, they soon return to the clothes. Moreover, these lice lay most, usually all, of their eggs in clothes rather than on the body. Third, the name clothes lice draws our focus to the clothes rather than the body. Treating clothes with insecticides, steaming clothes, and changing clothes regularly has been successful in reducing the prevalence and intensity of these lice over the past 100 years since World War I. Whether clothes lice and head lice are species, subspecies, or ecotypes (sensu bacterial ecotypes) is also controversial. We have previously reviewed the history of this controversy (*40*), and we will not repeat it here. We will use the name *P. humanus* for clothes lice and *P. capitis* for head lice because in our judgment, the evidence for these lice being separate species is overwhelming (*40*), regardless of which concept of a species of insect is used.

We studied the data of Leeson (*41*) and the subsequent interpretation and contextualization of this data by Buxton (*42*) and Busvine (*30*). Our laboratory colony of *P. humanus* lice was started in 2000 from eggs, nymphs, and adults of the Orlando strain of *P. humanus* lice. This strain was isolated from lice at the London School of Tropical Hygiene and Medicine that were derived from the Orlando strain that had been kept for many years at the US Agriculture Bureau of Entomology and Plant Quarantine (Orlando, FL, USA). The Orlando strain of *P. humanus* lice was adapted to feeding on the blood of rabbits (*43*,*44*).

## Killing Eggs of Clothes Lice by Holding Eggs at Suboptimal Temperatures <32°C

**Figure 3:**
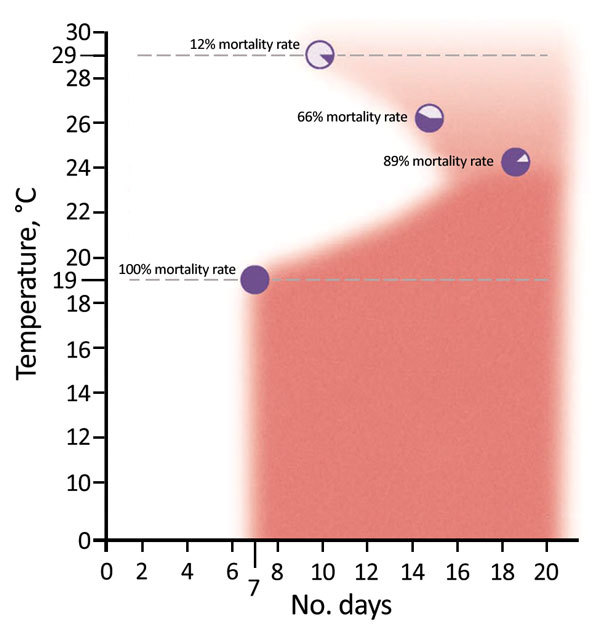
Mortality rates for the eggs of *Pediculus humanus* clothes lice, Ethiopia. Dark red shading indicates 100% mortality rate and light red shading <100% mortality rate for eggs. At 8°C–19°C, a total of 100% of eggs did not hatch; at 24°C, a total of 89% of eggs did not hatch (those that hatched took 14.5 days [range 13–19 days] to hatch); at 26°C, a total of 66% of eggs did not hatch; and at 29°C, a total of 12% of eggs did not hatch (88% hatched). Dotted lines denote 12% (top) and 100% (bottom) mortality rate temperatures. Data were obtained from Leeson (*41*).

Leeson (*41*) showed that 7 days at <19°C killed 100% of eggs of *P. humanus* clothes lice. At temperatures >19°C, the situation was more complicated, but the longer the eggs were held at temperatures lower than the ideal temperature for eggs (32°C), the more eggs died (*41*). We observed a complex relationship between temperatures >19°C and the proportion of eggs that did not hatch (i.e., 89% did not hatch at 24°C, 66% at 26°C, and 12% at 29°C) (Figure 3). Nonetheless, keeping eggs of clothes lice at virtually any temperature <32°C killed some of the eggs. The lower the temperature, the more eggs died. The longer eggs were held at suboptimal temperature, the more eggs died (*41*). Leeson (*41*) reported that favorable temperatures for *P. humanus* lice reared on the legs of humans were 30°C–32°C at a relative humidity of 65%. Our results were similar: 29°C–32°C at a relative humidity of 65% for the University of Queensland isolate of the Culpepper strain of *P. humanus* lice at the University of Queensland (S.C. Barker, unpub. data). The data of Leeson (*41*) showed that relative humidity does not have a substantial effect on the degree of death of eggs, although low relative humidity caused additional deaths of eggs (*41*).

## Killing Nymphal and Adult Clothes Lice by Starvation

**Figure 4:**
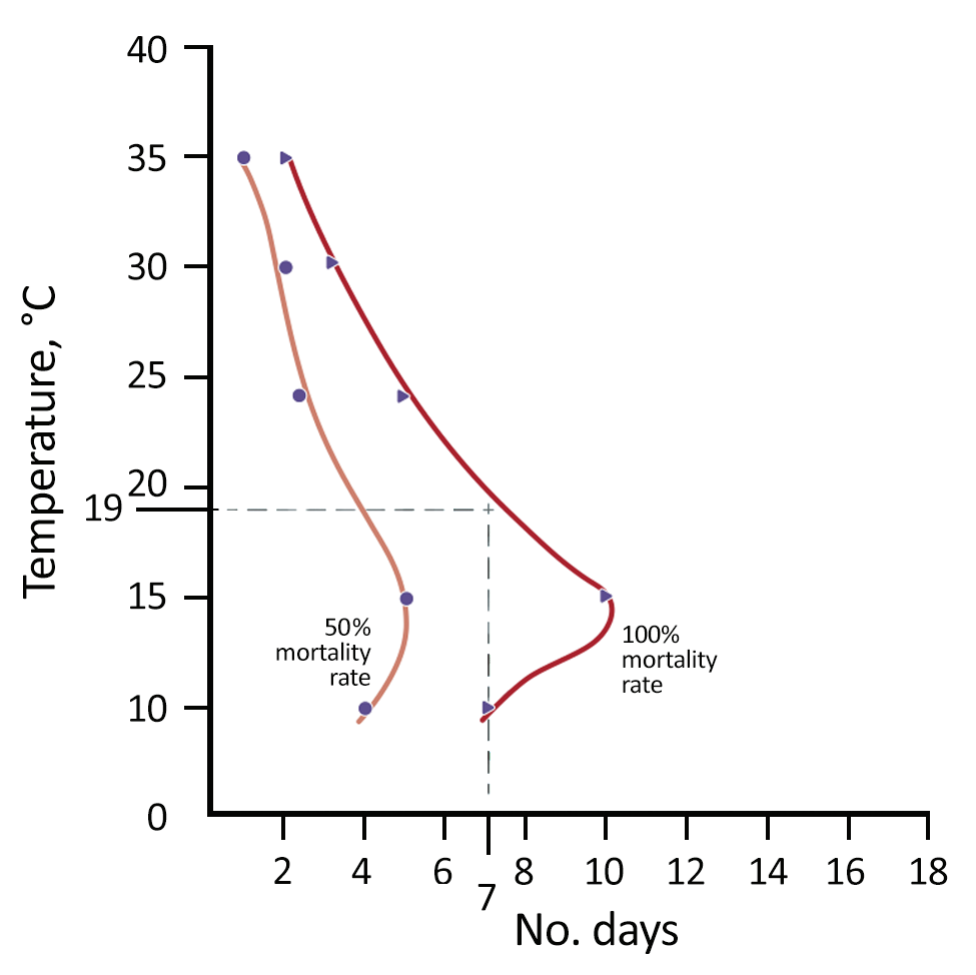
Number of days needed to starve to death adult and nymphal clothes lice (*Pediculus humanus*) at 5 temperatures (10°C, 15°C, 24°C, 30°C, and 35°C), Ethiopia. The mortality rate was 50% for lice after 5 days without a blood meal (i.e., away from the host) and 100% after 10 days without a blood meal, regardless of temperature. Dotted lines indicate temperatures of 0°C–19°C and days 0–7. Data were obtained from Buxton (*42*), and the figure was modified from Busvine (*30*).

Leeson (*41*) also showed that 100% of nymphal and adult clothes lice starved to death in 7 days at 10°C and in 10 days regardless of the temperature (Figure 4). Newly hatched nymphs are especially susceptible to starvation. If newly hatched nymphs at 32°C do not receive a blood meal within 48 hours of hatching, 100% will die (S.C. Barker, unpub. data, for the Barker isolate of the Culpepper strain of *P. humanus* lice). Even 24 hours without a blood meal will kill ≈50% of newly hatched nymphs (S.C. Barker, unpub. data, for the Barker isolate of the Culpepper strain of *P. humanus*). The lethal effect of high temperatures (>60°C) on clothes lice and their eggs is well known in Ethiopia and elsewhere. Healthcare clinics in Ethiopia and elsewhere often have 200-L steel steaming drums (Stammers Serbian barrels) (Figure 1) to kill clothes lice and their eggs in infested clothes during outbreaks of LBRF. Healthcare and medical students are instructed in the steaming drum method of killing clothes lice and their eggs in clothes and on blankets, which was first reported by Hunter in 1918 (*45*,*46*).

The lethal effect of low temperatures (<24°C) on clothes lice and their eggs is less well known. Clothes lice and their eggs depend on their human host. The long and intimate association between clothes lice and humans have led to clothes lice becoming completely dependent on their host for a warm environment and for food and water. In contrast to many other blood-sucking arthropods, eggs of clothes lice die in a few days at temperatures at little lower than optimum (Figure 3) (*30*). Moreover, clothes lice, especially newly hatched nymphs, easily starve to death (Figure 4) (S.C. Barker, unpub. data). Lloyd and Byam (*26*), in recounting their experiences as medical servicemen in World War I, emphasized constant changing of underclothing and believed that having 2 shirts rather than 1 helped reduce infestations with clothes lice. We extend this idea to all clothes, which in tropical climates, such as in Bahir Dah, means a shirt, underpants and shorts or trousers, and a jumper or coat. In our experience, all clothes in Bahir Dah, not just underclothes, might be infested with body lice.

## Potential Public Health Recommendations

If our strategy were successful, day laborers, street children, and live-in-church students in Bahir Dah might be encouraged to acquire and keep 2 sets of clothes rather than 1 set so that at any given time, 1 set of clothes might be kept in a plastic shopping bag for ideally 10 days to starve adult and nymphal lice and to kill (prevent hatching) eggs. Ten days in a plastic shopping bag at <19°C (e.g., on cold ceramic tile floors) should kill all eggs and lice in clothes. However, even 7 days should kill most eggs and lice (Figures 3, 4).

This hypothesis needs to be tested on clothes naturally infested with clothes lice and their eggs in Bahir Dah and elsewhere. Simple experiments on the effect of holding clothes away from hosts for 7 days and for 10 days might be conducted in boarding houses and church dwellings in Bahir Dah, and among day laborers of Bahir Dah and other areas in Ethiopia. For example, placing pieces of cloth (e.g., 20 cm × 20 cm) that were cut from naturally infested clothes that have only eggs into plastic shopping bags for 7 and 10 days would determine the effect of holding clothes away from hosts on hatching and development of nymphal lice from eggs in the cloth. All nymphal and adult lice would have been gently brushed away so that only eggs are left on the pieces of cloth. In addition, we would repeat this experiment with nymphal and adult lice that were brushed from infested clothes onto uninfested cloth (no eggs present). Likely limitations and complications to our strategy include the cost of acquiring a second set of clothes, that some lice might be displaced from clothes onto sleeping mats, and the perennial problem of reinfestation through contact with other infested clothes.

We note also the prospect of treatment with oral ivermectin to kill clothes lice. Oral ivermectin may be effective against head lice (*47**,**48*) and thus might also be effective against clothes lice. Therefore, oral ivermectin might be used alone or in combination with holding infested clothes away from hosts for 10 days to kill enough clothes lice to control LBRF in Ethiopia. However, a perennial problem with any kind of drug treatment is the cost, immediate and ongoing, to day laborers, street children, and yekolotemaries. In addition, resistance to ivermectin might develop in clothes lice.
